# Lactation counseling for maintaining exclusive breastfeeding in adolescent mothers: a trial protocol

**DOI:** 10.1186/s40814-021-00950-9

**Published:** 2021-12-16

**Authors:** Iliana Milena Ulloa Sabogal, Claudia Consuelo Domínguez Nariño, Mary Alejandra Mendoza Monsalve

**Affiliations:** 1 Universidad Industrial de Santander, Escuela de Enfermería, Bucaramanga, Colombia; 2 Joven Investigador Colciencias, Bogota, Colombia

**Keywords:** Breastfeeding, Nursing education, Pregnancy in adolescence, Nursing care, Nursing process

## Abstract

**Background:**

Adolescent mothers have lower rates of initiation, continuation, and exclusivity of breastfeeding, and even more so in the first pregnancy. Current interventions target adult women, and little evidence is available for breastfeeding promotion among adolescents.

**Methods:**

This is a pilot study protocol with a parallel, single-blind, randomized, and controlled trial design, to evaluate the feasibility of the intervention “Lactation Counseling” in first-time adolescent mothers to maintain exclusive breastfeeding in the first 6 months of life. The control group will receive routine education in prenatal care and prenatal and childbirth classes, the experimental group will receive additionally the intervention “Lactation Counseling”, for 4 weeks, both conducted by trained nurses. Feasibility outcome includes recruitment and dropout rates, and, pilot outcomes will be the exclusive breastfeeding rate and the breastfeeding knowledge. Measurements will be taken at baseline, post-intervention, and 2, 4, and 6 months after childbirth.

**Discussion:**

Exclusive breastfeeding rates could be increased in adolescent mothers through nursing counseling interventions that are previously structured and evaluated from their feasibility. This study will allow the evaluation of the feasibility of an intervention in low-income, Latin American population adolescents.

**Trial registration:**

ClinicalTrials.gov NCT04655846, Registered 7 December 2020.

## Backgraund

Breast milk is the natural and optimal source of nutrition for the newborn; it is associated with multiple health benefits [1, 2], providing nutrients, hormonal immunoactives, and microbiomes necessary for growth and development [3]. Furthermore, in the mother, it reduces the probability of breast and ovarian cancers, improves birth spacing, prevents type 2 diabetes, obesity, and hypertension, among other benefits [4, 5].

Countries have made efforts to protect, promote and support exclusive breastfeeding (EB). It is expected that by 2025 the prevalence of EB will be 50% [6], although globally only about 38% of children benefit from it [7]. The outlook is worse in developing countries, such as Colombia with a prevalence of 36.1% in children under 6 months, and only 52.2% of children 6 to 12 months continued to receive breastfeeding [8].

Exclusive breastfeeding is one of the most important cost-effective strategies in the prevention of maternal and infant morbidity and mortality. An estimated 823,000 deaths of children under 5 years of age and 20,000 deaths of women from breast cancer could be prevented each year if breastfeeding were a universal practice [2].

The optimal practice of breastfeeding in adolescent mothers is determined by a range of personal and cultural factors such as attitudes about breastfeeding, perceived benefit, knowledge, previous experiences, self-efficacy, and affective or social support. All these factors significantly influence the duration of breastfeeding in adolescent mothers [9].

The development of educational strategies that intervene in the factors that influence breastfeeding in adolescent mothers is key. Therefore, nursing must acquire a commitment and responsibility in the implementation of educational interventions, motivating the mother to generate positive thoughts and attitudes, as well as providing a support network and knowledge that contribute to the significant improvement of breastfeeding practices and indices of initiation, exclusivity, and duration [10–14].

The Global Breastfeeding Scorecard 2019 urges that primary care facilities offer individual advice on infant and child feeding, seeking the goal of 80% of countries whose health institutions provide this advice by 2030. It emphasizes the importance of improving access to qualified breastfeeding counseling by providing specialized guidance that helps to make informed decisions and overcomes difficulties for breastfeeding, focusing on new mothers who have the greatest lack of knowledge and confidence [15].

To carry out educational interventions, the nurse must implement the nursing care plan, which provides patient-centered care and improves the expected results. To implement this care plan, the use of the Nursing Intervention Classification (NIC) [16] is recommended, which includes the nursing intervention “Lactation Counseling”; however, available evidence on the use of the classification in clinical trials is scarce especially in the adolescent maternal population [17].

## Purpose

This article describes the methodology of intervention design based on the nursing intervention “Lactation Counseling” that aims to determine the feasibility of this intervention in first-time adolescent mothers for maintaining exclusive breastfeeding in the first 6 months of child life.

As secondary objectives, we expect to estimate the recruitment and dropout rates, and the potential effectiveness of this intervention in the exclusive breastfeeding rate and breastfeeding knowledge.

## Methods

### Design

**Fig. 1:**
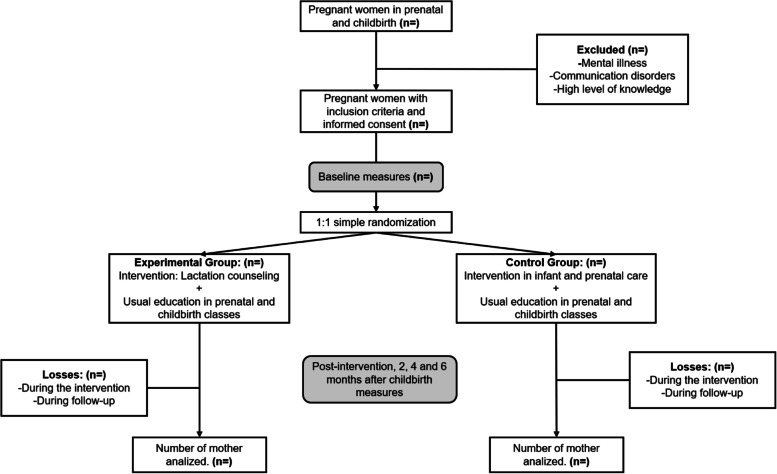
Flowchart of the study protocol

This is a pilot study protocol for a randomized controlled trial using a parallel-group design with a 1:1 allocation ratio, to evaluate the preliminary effect on exclusive breastfeeding rates of the intervention “Lactation Counseling” described in the Classification of Nursing Interventions (NIC) (16) (*n* = 59), versus usual education and nursing interventions not related to breastfeeding (*n* = 59) in first-time pregnant adolescents, attending the control prenatal, and followed-up with for up to 6 months after delivery (Fig. 1).

### Participants

Eligible participants are first-time teenage mothers; between 14 and 19 years old, between 20 and 30 weeks gestation, and with the native Spanish language. Adolescents with psychiatric or communication disorders will be excluded and who have obtained a score greater than or equal to 3.8 on the Knowledge outcome: Breastfeeding (1800), described in the Nursing Outcomes Classification (NOC) [18].

### Settings and locations

The study will be carried out in two public hospitals in the cities of Girón and Piedecuesta in Santander, Colombia, which mainly provide care for low-income people. The recruitment of participants began in 2019 and is expected to end in 2021 due to delays caused by quarantines and restrictions to control the COVID-19 pandemic. The protocol is approved by the research ethics board of the Industrial University of Santander and is covered by the inter-institutional agreement between the participating hospitals; it has obtained approval and funding from the university’s Research and Extension Directorate (#2453) and additional funding from young research program form the Ministry of Science, Technology, and Innovation (#8009).

Pregnant women attending prenatal controls will be invited to participate in the study. Pregnant women who meet the inclusion criteria and sign informed consent will fill out a questionnaire designed based on the Knowledge outcome: Breastfeeding (1800). Adolescents who obtain a score on the knowledge test less than or equal to 3.8 points will be invited to participate in the intervention. Pregnant women scoring less than or equal to 3.8 in breastfeeding knowledge will be invited to participate as they are susceptible to increase breastfeeding knowledge.

### Interventions

#### Control group

This group will receive the usual education given in prenatal control and maternity preparation classes, using the interventions: Infant Care (6820) and Prenatal Care (6960), taken from the Nursing Interventions Classification [16]. This group will not receive specific interventions in breastfeeding and the information on this aspect will be that which is usually offered in prenatal control.

This group will receive the interventions in four educational sessions with the following topics: physical-psychological changes and care during pregnancy, the process of labor, delivery, and postpartum in its different stages, care of the newborn at home, and family planning. The sessions will be carried out in groups of a maximum of ten pregnant women, led by a nurse trained in maternal and perinatal care. The frequency of each session will be weekly, lasting between 45 and 60 min each. The sessions will be supported by audiovisual materials like slides, photography, videos, and educational games.

#### Experimental group

In addition to the usual education given in prenatal control and maternity preparation classes, this group will receive the intervention “Lactation Counseling” (5244) defined as “assisting in the establishment and maintenance of successful breastfeeding” [16].

The intervention will consist of four educational sessions with seven topics (see Table 1). The frequency of each session will be weekly, lasting 45–60 min each. Each session contains three parts: (1) introduction to the topic where the objective of the session is explained and the pre-knowledge and usual practices in the community on the topic are explored, (2) development of the topic using PowerPoint presentations, audiovisual material such as photographs and videos, simulation material as well as strategies to encourage the participation of mothers such as games and challenges, and (3) end of the session reviewing the main lessons, addressing doubts and concerns, and carrying out an evaluation with questions on the topic of the session.

**Table 1:** Summary description of educational sessions for Lactation Counseling intervention

Session	Content	Methodologies	Materials
1	• Composition of breast milk • Psychological and physiological benefits of breastfeeding	• Lecture method • Audiovisual Presentation • Group assignment	PowerPoint presentations Breast milk production video Breastfeeding benefits video Breastfeeding kit with educational elements such as games and models (bottles with types of breast milk) Explanatory sheets with each of the components of breast milk. Sheets with text to identify the benefits of the breast for the mother, baby, family, and society.
2	• Technique and positions for breastfeeding	• Audiovisual Presentation • Demonstration • Practical exercise	Simulated models (baby dolls) Video on breastfeeding and nipple latch technique
3	• Extraction, conservation, and administration of breast milk	• Lecture method • Audiovisual Presentation • Demonstration • Practical exercise	PowerPoint presentations Video on storage, conservation, and administration of breast milk. Vest-type simulators for breast milk extraction
4	• Breast complications during breastfeeding • Lactating mother needs (rest, hydration, and a balanced diet)	• Lecture method • Audiovisual Presentation • Demonstration • Practical exercise • Group activity	PowerPoint presentations Breastfeeding kit with educational elements such as games and models (breast models) Lottery game on care and complications during breastfeeding Memory game on nutrition and hydration during breastfeeding Video on care for the prevention of breast complications

Participants will not receive educational material about interventions, neither will they be encouraged nor prohibited from seeking information about breastfeeding by other means. As a retention strategy, all mothers will be phone-called before each session to encourage their attendance and another phone call will be made during the first week after delivery to know the health status of the mother-child binomial.

### Outcomes

The primary results will be oriented to evaluate feasibility in the implementation of the counseling intervention concerning the proposed times for intervention and the difficulties that may arise in the application of these, and the recruitment and dropout rates.

Secondary outcomes will be the exclusive breastfeeding rate in the first 6 months postpartum. We use the WHO definition of exclusive breastfeeding: “exclusive breastfeeding for at least 6 months in all infants,” meaning that the infant receives only breast milk and no other liquids or solids, not even water, except for oral rehydration solution or drops/syrups of vitamins, minerals, or medicines [19].

The previous outcome will be evaluated by home visits at 2, 4, and 6 months postnatal, and a checklist will be used to verify the adherence and maintenance of mothers to breastfeeding by asking the following: offer exclusive breastfeeding, offer food or fluids other than breast milk, ensure breastfeeding even when separated from your child (expressing breast milk), describe correct breastfeeding technique, and recognize the importance of breastfeeding for the growth and development of the newborn. Additionally, mothers will be invited to continue breastfeeding and participating in the study.

Another secondary outcome will be the level of knowledge about the breastfeeding process using a questionnaire (Table 2), based on the nursing Knowledge outcome: Breastfeeding (1800) defined as the “Extent of understanding conveyed about lactation and nourishment of an infant through breastfeeding” [18]. For questionnaire design, we select 7 indicators and construct a statement with multiple-choice answers for each one. For the evaluation, a Likert-type scale will be used with a score of 1 to 5 (1, no knowledge; 2, little knowledge; 3, moderate knowledge; 4, substantial knowledge; and 5, extensive knowledge). The internal consistency of the questionnaire was 0.8712 Cronbach’s alpha coefficient.

**Table 2:** Items and indicators to quantify the nursing knowledge outcome: breastfeeding (1800)

Indicator 180003: composition of breast milk, milk outlet process, and initial vs late milk	
1. Occurs between 5 and 10 days after delivery	
2. Contains less sugar, fats, and vitamins of B complex and vitamin C	
3. Contains proteins, sugar, fats, minerals such as sodium, calcium, iron, selenium, zinc, and B complex vitamins and vitamins such as C, A, E, K.	
4. Its composition contains between 88% and 90% water	
5. Occurs in the first 3 to 4 days after delivery	
6. It is yellow, thick and of little amount	
7. Contains more sugar, fats, calories, and B Complex vitamins and vitamin C	
8. It occurs progressively up to about 100 ml a day	
9. Contains fewer proteins, antibodies, and vitamins A, E, K	
10. It occurs in an amount of 400 to 600 ml per day	
11. Occurs from the tenth day after delivery	
12. Contains more protein, minerals such as sodium, iron, selenium, zinc, and vitamins such as A, E, K	
13. Its composition contains 87% water	
14. It is produced in an amount of 700 to 800 ml per day	
Indicator 180001: benefits of breastfeeding	
15. Prevents breast, uterine, and ovarian cancer	
16. Prevents respiratory diseases such as bronchitis, pneumonia, and gastrointestinal diseases such as diarrhea and dehydration	
17. Prevents the onset of diseases such as allergies, obesity, high blood sugar, high blood pressure, and cancer	
18. Prevents postpartum depression states (mood affecting women after giving birth, characterized by feelings of extreme sadness and anxiety)	
19. Prevents constipation and cramping	
20. Prevents child malnutrition	
21. Facilitates the affective bond between mother and child	
22. Strengthens self-appreciation, self-confidence, and emotional satisfaction	
23. Contributes to postpartum weight loss	
24. Lowers the risk of osteoporosis (a disease that thins and weakens bones, causing them to break easily)	
25. Promotes better growth and development of physical, language, and social capacities	
26. Reduces risk of postpartum hemorrhaging or bleeding	
27. Lowers the risk of heart disease	
28. Improves intelligence	
29. Delays the return of ovulation and menstruation	
Indicator 180005: proper technique for breastfeeding the baby	
30. The mother should select the most comfortable position	
31. The baby's body must be attached to the mother's body	
32. The baby's head and body are in the same direction	
33. The mother should grab the breast with her C-shaped hand (placing her thumb above the breast and the other four fingers below the nipple and behind the areola (ring of pigmented skin around the breast)	
34. The mother should bring the child closer to the breast and not the breast to the child	
35. The mother should stimulate the baby to have a search reflex, bringing the nipple closer to the baby's lip and when it opens its mouth to insert the nipple and the areola	
36. The baby's lips should remain outside like the mouth of a fish	
37. The baby's mouth should coat the entire areola (dark area of the breast)	
38. The baby's chin must touch or almost be touching the mother's chest	
Indicator 180006: adequate position of the infant during breastfeeding	
39. Stretched or cradle position (classic or traditional)	
40. Cross-cradle position	
41. Parallel stretched position (side lying down)	
42. Rugby ball or football position (inverted)	
43. Sitting or horse position	
44. Face-up position	
45. Vertical or standing position	
Indicator 180014: signs of mastitis, duct obstruction, and nipple trauma	
46. Breast skin is shiny, red, tense, and warm	
47. Formation of a mass or lump in the breast that is palpable and painful, often with redness of the skin in that area	
48. Painful cracks or wounds on the nipple	
49. Uncontrolled fever, chills, general discomfort, nausea, vomiting, headache	
50. Formation of a mass or lump in the breast, severe pain, swollen and hardened sinus, obstruction at the milk outlet (milk does not flow), pus secretion	
51. Inflammation or swelling in the breast with a feeling of warmth	
52. Nipple pain	
53. Cracks or nipple wounds that may bleed	
54. Decreased milk flow	
55. No fever or symptoms of general discomfort	
Indicator 180015: appropriate techniques for the extraction and storage of breast milk	
56. Massage into the breast before breast milk extraction	
57. At room temperature: breast milk is preserved for up to 4 h, it is advisable to leave it in a dry place, protected from sunlight and heat.	
58. In the freezer: breast milk is kept frozen for up to 15 days, so it is recommended not to constantly open and close the freezer. If the refrigerator is two doors, breast milk can be stored for up to 3 months.	
59. With your hand in the shape of a C, place it on the breast (placing your thumb above the breast and the other four fingers below the nipple and behind the areola (dark area of the breast)	
60. In a refrigerator or refrigerator: breast milk is stored for up to 12 h, it is advisable not to place it in the refrigerator door to avoid temperature changes every time the door is opened or closed.	
61. Lean the body forward, squeeze the breast without swiping your fingers, and gently push against the ribs	
62. Then move your fingers forward and repeat the procedure as many times as necessary simultaneously and smoothly	
Indicator 180020: need for fluid intake by the mother	
63. It is advisable to increase the consumption of liquids, preferably water, as it is the largest component of breast milk	
64. Water intake during lactation should be sufficient to compensate for the loss of water through milk	
65. Vitamin C-rich juices are recommended	
66. The most recommended liquids for women during breastfeeding are water, fruit juices, and milk	
67. Avoid drinking alcoholic beverages, excessive coffee, and tea (more than 2 cups a day, during the term of breastfeeding)	

At the end of the intervention, the level of knowledge acquired will be verified and a home visit will be made at 2, 4, and 6 months after the birth of the babies. The questionnaire will be used again in each of the visits to evaluate the level of knowledge over time and a checklist will be used to verify the adherence and maintenance of mothers to breastfeeding.

### Sample size for secondary outcomes

We used STATA-12 to calculate the sample size with the following statistical parameters: a rate of exclusive breastfeeding of 70% in the experimental group and 40% in the control group, a power of 80%, an alpha error of 5%, and the rate of abandonment of exclusive breastfeeding being 2.5 times higher in the control group compared to the experimental group. Additionally, for the knowledge level outcome, we considered a delta or expected difference of 0.4 in the knowledge assessment outcome score between both groups, a power of 95%, an alpha of 5%, a standard deviation of the outcome scores of 0.5, an average of correlations between the first and second assessment of 0.5. Finally, the sample size estimated was *n* = 118 primigravidae mothers including 20% of the possible losses; distributed by simple randomization with a ratio of experimental group/control group 1:1 (*n* = 59 pregnant women in the experimental group and *n* = 59 pregnant women control group).

### Randomization and blinding

We will use simple randomization with an allocation ratio of 1:1. The generation of the allocation sequence will be carried out using a succession of true random numbers stored in tables of random digits. A person who does not know the participants will access this table to find out which group each participant belongs to, depending on whether the number was odd or even, and will inform the nurses who will carry out the intervention. The nurse who will perform the intervention “Lactation Counselling” will only maintain contact with the experimental group, and the nurse who will carry out the initial and final measurements along with the person who will perform the data analysis will be blinded to the group assignment. The nurses conducting the interview and statistical analysis of data will be masked in group assignments. To avoid contamination of the participants between the control group and the intervention group, they will meet on different days and times to attend the activities of the interventions.

### Ethical consideration

This research is based on the World Medical Association Declaration of Helsinki and the research guidelines in Colombia (Resolution 008430/1993 and Law 911/2004) and has the approval of the Ethics Committee in Scientific Research of the Industrial University of Santander. The parents of the participants must sign the informed consent, as well as a consent for those under 18 years of age because they are considered a population of minors.

### Statistical analysis

The information will be recorded in EpiData 3.1. An intention-to-treat analysis will be performed. We will use descriptive statistics: continuous variables will be presented using median and standard deviation after evaluation of the normal distribution of the variable, otherwise, median and minimum, and maximum values will be used. The categorical variables will be presented in absolute frequencies and relative in percentages asymmetrical. Categorical data will be summarized as counts and percentages. The categorical and continuous variables will be compared according to the treatment group using chi-square tests or Fisher’s exact tests and Student's *t* test or Mann-Whitney's *U* test, respectively. The exclusive breastfeeding rate will be calculated at 2, 4, and 6 months of follow-up with their respective confidence intervals. To evaluate the preliminary effect of the intervention on the level of Knowledge: Breastfeeding, two approximations will be made by different statistical methods: mean differences through independent samples Student's *t* test and repeated measures ANOVA with their respective confidence intervals. Additionally, the effect of the intervention on the abandonment of exclusive breastfeeding will be calculated in terms of relative risk using binomial regression.

## Discussion

We detail the design of a nursing intervention to standardize breastfeeding counseling, as well as the evaluation of its effect on adolescent mothers. This intervention has the advantage of having been designed using standardized language from the Nursing Intervention Classification and using the same standardized language to evaluate its effect through the Nursing Outcome Classification, using standardized language facilitates the use of interventions and the measurement of their effect in different populations, as well as consolidating the use of a specific nursing language.

Educational interventions in first-time mothers have shown effectiveness in maintaining exclusive breastfeeding in the first 6 months of life [13] and an increase in the level of knowledge about breastfeeding [20, 21]. However, these interventions were not carried out on adolescent mothers, although pregnancy at this stage of the life cycle is considered a global public health problem is given that nearly 16 million adolescents give birth every year in the world, which is equivalent to 11% of all births worldwide, a figure that increases in developing countries. Likewise, Latin America and the Caribbean have the second-highest adolescent fertility rate in the world, estimating 67 births per 1000 girls between the ages of 15 and 19 between 2010 and 2015 [22]. The above figures denote the urgency of providing counseling support to sustain breastfeeding in these young mothers, with appropriate methodologies for these ages.

Additionally, it should be considered that the adolescent mother is generally not prepared to carry out effective breastfeeding, and in developing countries, it is working mothers who can put breastfeeding at risk. Different studies have established that adolescent mothers, compared to adult mothers, are less likely to initiate breastfeeding and those who initiate it are more likely to abandon it. Among the reasons for dropping out, causes such as hypogalactia, returning to school, medical indication, causes related to the nipple, maternal illness, mother work, and acid reflux in the newborn have been described [23]. In a study carried out in adolescent mothers, 39.4% lactated for 6 months and 9.8% lactated for more than 6 months; the reasons why they do not feed their child with only breast milk are 9.5% produce little milk, 12% the child is left hungry, and 20.7% other causes [24]. On the other hand, a study in South Africa found that adolescent mothers knew the benefits of breastfeeding but abandoned it easily due to its lack of practicality when faced with the need to go to school [25] a finding similar to that found by Acosta Silva in Ecuadorian adolescents [26], adding as a barrier the insufficient breastfeeding education during prenatal or in the early postpartum period [26, 27]. The foregoing highlights the importance of designing interventions that promote exclusive breastfeeding and therefore evaluating its effect.

On the other hand, a review by Lumbiganon et al. in 2016 found that the majority of intervention studies to promote exclusive breastfeeding had been done in high-income countries [28]. This is a pilot study that seeks to know the efficacy of a nursing intervention in low-income adolescent mothers in a developing country, considering that sustaining breastfeeding depends on social and cultural factors and given the need to explore an intervention that is low cost and easy to perform by nurses.

Even though this pilot uses a randomized design, to prevent potential biases, some measures will be taken to reduce their appearance: offer the possibility of participation to all adolescents in maternity classes, have strict measures for the random assignment of mothers to groups. Ensure blinding of nurses who will assess the outcomes and those who analyze the data. It is worth highlighting the importance of the intervention activities being standardized and supervised with prior training of the nursing staff.

It is important to note that mothers will be able to access information or training on breastfeeding in a complementary way through different means and could bring the levels of knowledge about breastfeeding closer to those of the intervention group. There can also be potential contamination of the groups if some mothers who are into different intervention/control groups have a close relationship given the proximity of their homes and the similarity in age and share the learning and experiences acquired during the interventions.

This study, given the nature of the intervention, also provides a higher level of scientific evidence, which will undoubtedly support the advancement and training of professionals in the discipline of Nursing, by basing knowledge and thinking to take actions and decisions that form part of the practice and assistance of nursing care in maternal and child health.

We consider that this study is feasible and replicable, taking into account as participants mothers who already attend prenatal care or and birth classes, which are programs that are usually offered to mothers. Likewise, agreements between research entities and health care centers can help improve the feasibility of this study. In addition, the health services have nurses who can make home visits and phone calls or assign consultations for counseling or follow-up.

Finally, the findings of this study will be a valuable resource for the management of nursing care, which will support the formulation, implementation, monitoring, and evaluation of national policies aimed at the promotion, support, and protection of breastfeeding, by involving the educational component as an important strategy that guarantees respect and protection of the act of breastfeeding as a human right appropriate for women and their children.

## Data Availability

The datasets generated and analyzed during the current study will be available from the corresponding author on reasonable request.
